# Structural lubricity in soft and hard matter systems

**DOI:** 10.1038/s41467-020-18429-1

**Published:** 2020-09-16

**Authors:** Andrea Vanossi, Clemens Bechinger, Michael Urbakh

**Affiliations:** 1 CNR-IOM Democritos National Simulation Center, Trieste, Italy; 2 International School for Advanced Studies (SISSA), Trieste, Italy; 3 Fachbereich Physik, Universität Konstanz, Konstanz, Germany; 4 School of Chemistry and The Sackler Center for Computational Molecular and Materials Science, Tel Aviv University, Tel Aviv, 6997801 Israel

**Keywords:** Soft materials, Theory and computation, Surfaces, interfaces and thin films

## Abstract

Over the recent decades there has been tremendous progress in understanding and controlling friction between surfaces in relative motion. However the complex nature of the involved processes has forced most of this work to be of rather empirical nature. Two very distinctive physical systems, hard two-dimensional layered materials and soft microscopic systems, such as optically or topographically trapped colloids, have recently opened novel rationally designed lines of research in the field of tribology, leading to a number of new discoveries. Here, we provide an overview of these emerging directions of research, and discuss how the interplay between hard and soft matter promotes our understanding of frictional phenomena.

## Abstract (web summary)

Structural lubricity is one of the most interesting concepts in modern tribology, which promises to achieve ultra-low friction over a wide range of length-scales. Here the authors highlight novel research lines in this area achievable by combining theoretical and experimental efforts on hard two-dimensional materials and soft colloidal and cold ion systems.

## Introduction

Despite the fundamental and practical relevance of dissipative interface phenomena and its long history in science, the complexity of the highly out-of-equilibrium processes across confined sliding interfaces still leaves several key aspects of tribology not well understood^1–3^. Setting aside empiricism and engineering, scientists embracing distinct—yet complementary—experimental, theoretical, and computational approaches, have recently made crucial systematic inroads in the atomic-level physics involved in frictional phenomena. Spanning several decades of the involved scales of length, time, and energy, the ubiquitous nature of friction, together with a remarkable number of material-independent features, suggests indeed potentially fruitful links among physics, chemistry, materials science, soft matter, geology, and other fields of past and current research and technology, making tribology a pretty unique subject.

On the wave of recent advances and achievements in the field, we aim at providing, via this Perspectives article, a glance at some topics which in our view contain potential seeds for further work, development and new connections. Specifically, out of serendipity, two very distinctive operative frameworks have recently brought about promising avenues of research and new discoveries in tribology: the friction-related physics of hard two-dimensional (2D) layered atomic materials and that of optically and topographically trapped soft microscopic systems, such as cold ions and colloids.

**Fig. 1:**
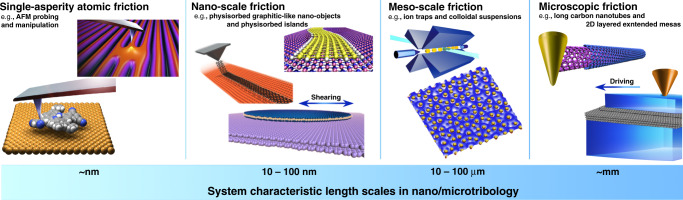
Systems and length scales of nano/micro tribology: from single-asperity atomic contacts to microscopic sliding interfaces. The multifaceted discipline of tribology starts, at the shortest scale, by the investigation, via proximal-probe techniques, of a single atomistically small contact as that realized, e.g., between a sharp atomic force microscope tip, or a deposited molecule (reprinted (adapted) with permission from ref. ^144^. Copyright (2016) American Chemical Society), and an underlying reference substrate. Entering into the nanoscale range of tens-of-nanometer extended adsorbates, such as AFM-manipulated graphitic ribbons (reprinted (adapted) with permission from ref. ^30^. Copyright (2018) American Chemical Society) and metallic clusters, or rare-gas islands sheared by a quartz-crystal microbalance, interface geometry and incommensurability features rule the frictional response. With unprecedented real-time resolution processed at the single-particle level, the novel experimental mesoscale techniques of driven trapped cold ions and colloids have recently made crucial systematic inroads in the physics of frictional phenomena. State-of-the-art technology and fabrication procedures, especially in the realm of 2D layered materials (e.g., graphitic tubes and mesas), have nowadays offered tribology the chance to investigate atomistically well-characterized mechanical contacts up to the millimeter range and beyond. With the exponential boost of computer power in the last decades, numerical modeling and atomistic simulations are jointly advancing our theoretical understanding across all these length scales.

At microscopic scales (Fig. 1), where mechanical contacts are generally much better characterized compared to the irregular interface between macroscopic objects, hard and soft matter can remarkably meet with a view to understanding and exploiting the way frictional forces and energy dissipation mechanisms do depend on the relative geometry and commensurability of the sliding interface. This scenario underlies one of the most theoretically fascinating and technologically important concepts of modern tribology, i.e., structural superlubricity, a sliding state of vanishing friction.

## Superlubricity and 2D atomic materials: observation, robustness, and demise

There are many alternative strategies for achieving ultralow friction^4,5^. The most common example is lubricated friction, where lubricant additives, polymer brushes, ionic liquids, or hydration layers are used to provide ultralow friction and remarkable durability of interfaces^6–9^. Notably, ultralow friction can be achieved also in the absence of such lubricants. Thus, amorphous carbon coatings can provide ultralow friction coefficients^10–13^, and they are already used in industrial applications, including razor blades, magnetic hard drives, microelectromechanical systems and more.

**Fig. 2:**
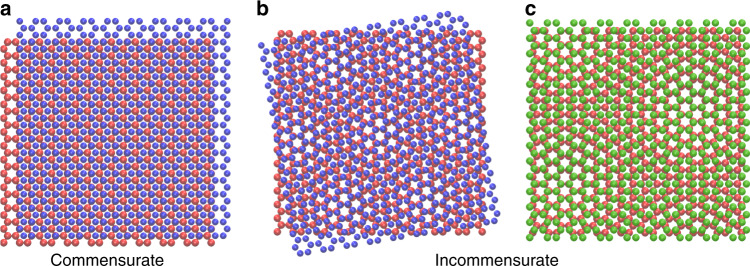
Geometrical configurations at crystalline interfaces. Mating identical surfaces with the same lattice spacing (red and blue) may give rise to both **a** commensurate interfaces when aligned and **b** incommensurate ones when orientationally misaligned at a misfit generic angle. **c** Structural incommensurability may also emerge when dealing with crystalline lattice-mismatched surfaces (red and green). The overall geometrical features of the contacting interface are captured in terms of the superstructure of the moire’ interference pattern.

When dealing with well-defined mating interfaces, the idea of nearly frictionless sliding, in both soft and hard matter contacts, relies on the possibility to predict the physical response of a system under shear based on interface geometrical features more than upon distinct system-dependent characteristics. Structural incommensurability, arising from surface lattice mismatch or misalignment as shown in Fig. 2, can prevent interlocking and collective stick-slip motion of interface asperities, with a consequent vanishingly small frictional force. Beyond the intrinsic interest for basic science research, the quest and design of superlubric materials constitute a subject of practical importance in nano- and micro-mechanics, aimed to significantly reduce the friction, energy dissipation and wear in hi-tech devices functioning at various length scales.

### A route toward frictionless and wearless sliding: theoretical prediction and nanoscale experimental observation

Aubry’s pioneering study of the Frenkel–Kontorova (FK) class of models^14–18^ in the physical context of frustrated incommensurate structures and its connection to the renowned Kolmogorov–Arnold–Moser framework of the onset of chaos in dynamical systems^19,20^, is cast as one of the deepest achievements in recent theoretical mechanics and dynamical systems theory.

Aubry theory leaves nowadays the elegant mathematical realm of number theory and Hamiltonian maps to become practically relevant and cornerstone of the physical understanding of many length-scale competition phenomena in complex systems, as can be found, e.g., in both condensed matter and soft-matter physics. Particularly, the surprising existence of a possible frictionless sliding regime, today a pervasive nano/mesoscale tribological concept called superlubricity and first named by Hirano and Shinjo^21–23^, can be rigorously drawn in the framework of the standard one-dimensional (1D) FK model, describing a chain of nearest-neighbor interacting particles (the slider) subject to a corrugated periodic potential (the underneath substrate)^24,25^. According to Aubry, in the thermodynamic limit, an incommensurate 1D chain/substrate interface undergoes a sharp remarkable transition at a critical value of the substrate potential corrugation between a lubric frictionless dynamics and a pinned frictional sliding. In other words, the static friction, i.e., the minimal force required to initiate motion, should vanish, provided the two mismatched mating surfaces are stiff enough compared to their mutual interaction; in addition, under such conditions, the incommensurate contact geometry promotes also an ultralow dissipative dynamical regime during sliding.

Even though, at first glance, Aubry’s mathematically rigorously proven scenario may seem quite academic when compared to the intrinsic 2D (or even 3D) complexity of a microscopic sliding interface, structural superlubricity has indeed started to be observed in an increasing number of recently implemented experimental cases.

Back in the nineties, few proximal-probe based studies provided the first experimental evidence of superlubricity^22,26–28^. In 2004, one groundbreaking experiment in the field demonstrated interlayer registry-dependent friction in a nanoscale contact between a pristine graphite surface and a graphite flake attached to an AFM tip^29^. There, it was shown that when the atomic lattices of the two contacting surfaces are aligned in registry, high interlayer friction values are measured, while when orientationally misaligned friction practically vanishes. This observation first marked layered materials as promising candidates for dry friction applications in nano/microelectromechanical systems (NEMS/MEMS), as well as improved lubricants or lubrication additives for more macroscopic devices.

Still focusing on nano- up to mesoscale experimental systems, other works, including nanomanipulation of deposited adsorbates (in the form of nanoclusters and 2D graphite-like patches)^30–40^, telescopic dynamics and water flow in nanotubes and confined geometries^41–43^, rare-gas island inertial motion^44–46^, sliding colloidal layers and ion-crystal simulators^47–53^, and transport in quasi-1D Wigner solids^54,55^, have recently uncovered signatures of a superlubric behavior.

With the potential of controlling dissipation and reducing wear in high-performance mechanical devices functioning at various scales, all that has brought about great fundamental and technological interest toward the nature of structural superlubricity, its robustness, the feasibility of scaling it up, and the possible mechanisms leading to its failure and breakdown. The search and design of novel ultralow frictional interfaces is a timely subject of practical importance. Specifically, the main challenges today are to achieve robust superlubricity^4,56–59^, sustainable against external load, high velocities, ambient conditions and varying contact geometries, and to upscale this phenomenon to microscopic and eventually macroscopic system length scales.

### Robustness and scale up: the realm of 2D atomic materials and heterojunctions

As prescribed by Aubry’s theory, dry solid-on-solid contacts displaying vanishing low values of static and kinetic friction, arising from effective cancellation of lateral force components, are characterized by key ingredients, such as interface incommensurability, intrinsic rigidity of the mating surfaces, paired with mutual weak interactions. Within this framework, the class of 2D layered materials^60^, where weak van der Waals (vdW) forces couple extremely in-plane stiff atomic layers, does emerge as an exemplary candidate.

**Fig. 3:**
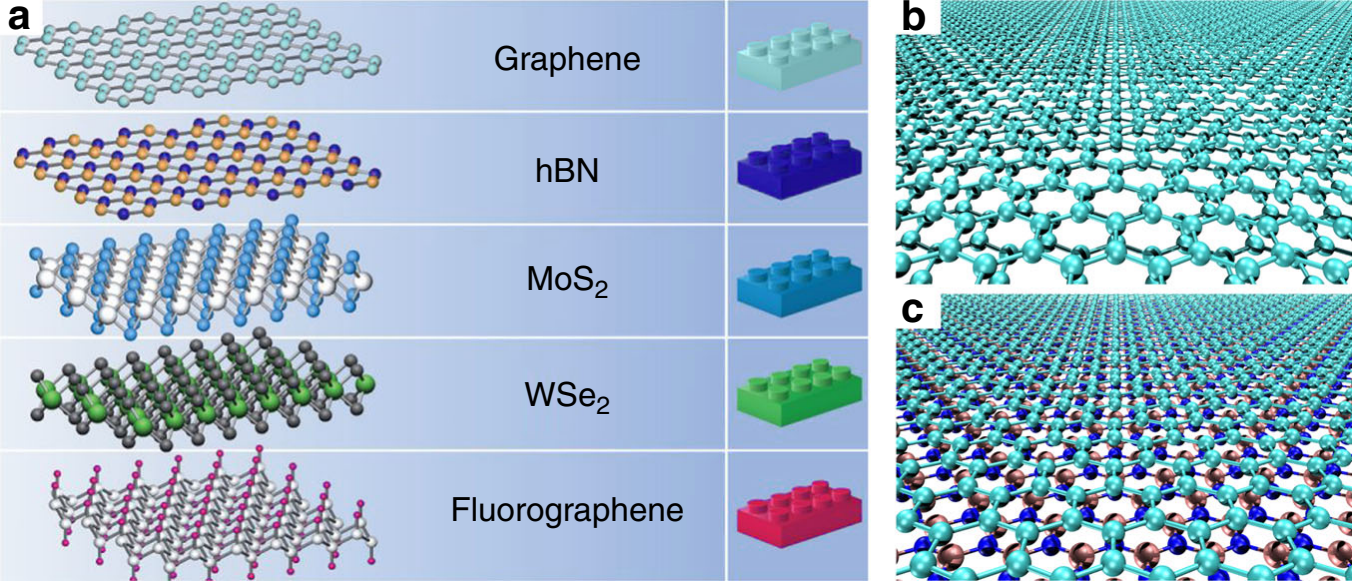
2D materials as building blocks for clean and atomically flat mechanical junctions. **a** Examples of 2D materials used in the construction of layered van der Waals structures (adapted from ref. ^61^). **b**, **c** Homogeneous—graphene on graphite—and heterogeneous—graphene on hexagonal boron nitride—interface junctions, respectively, characterized by weakly coupled and extremely stiff atomic layers, and thus exploited for mechanical and tribological applications. While structural lubricity requires an angular misalignment for the homogeneous contact (**b**), the hybrid junction (**c**), thanks to the intrinsic lattice incommensurability, ensures superlubric sliding^68–70^ independently of the surface relative orientation.

Indeed, the number of materials that can be thinned down to single layers has been widely increased since the first isolation of graphene monolayers (Fig. 3). Mechanical exfoliation techniques and subsequent combination via stamping methods makes it indeed possible to create clean, chemically inert, and atomically flat structures of almost arbitrary layer arrangements^61^. Prototype examples for mechanical and tribological applications are 2D layered materials such as graphite, hexagonal boron nitride (*h*-BN), molybdenum disulfide (MoS_2_), and other dichalcogenides. Structural anisotropy of these elements, characterized by strong intralayer covalent bonding and weaker interlayer dispersive interactions, results in low interlayer friction and wear resistance. This state of affairs makes 2D materials promising candidates for achieving structural superlubricity in realistic mechanical systems. However, realizing this potential still requires a solution of many demanding problems standing in the way of scaling up of superlubricity to macroscopic contacts under ambient conditions.

Considerable progress in upscaling superlubricity was achieved in 2012 when this phenomenon was found for graphite samples of micrometer size^62–64^. Importantly, the superlubric behavior was observed to sustain under ambient conditions and at high sliding velocities up to 25 m/s^65^. Besides, measurements carried out in microscale graphitic junctions of different sizes, showed a power-law scaling of kinetic friction with the contact area, and found an exponent of 0.35 confirming the incommensurability of superlubric contacts^63^. The next step toward macroscale superlubricity was attained using centimeter-long bichiral double-walled carbon nanotubes that exhibit the intrinsic interwall incommensurability^66^.

One of the major obstacles in realizing robust superlubricity at homogeneous graphite-like contacts is their possible spontaneous reorientation, locking them back into commensurate high-friction configurations^67^. To overcome this issue, heterogeneous junctions (heterostacks) formed, e.g., between graphite and hexagonal boron nitride (*h*-BN) have been suggested theoretically^68–70^, predicting that even for aligned contacts the heights of potential barriers toward sliding significantly decrease above a certain contact size. This phenomenon results from the intrinsic mismatch of the interlayer lattice periodicities in hetero contacts, which leads to the formation of moiré superstructures, and to a transition from stick-slip motion to smooth soliton-like sliding with increasing contact size^70^. Thus, ultralow friction should be observed even for aligned heterojunctions, and dynamical surface reorientations will not eliminate superlubricity. Recent experiments supported these predictions, demonstrating superlubricity in the microscale heterogeneous contacts of graphite and hexagonal boron nitride for all mutual surface orientations^71^. Furthermore, the heterogeneous contacts may exhibit ultralow friction for significantly higher normal loads, than the homogeneous ones^70^. These experimental and theoretical findings show that van der Waals heterostructures hold great promises for dry tribological applications, which require ultralow friction and wear.

Less ideal forms of interface geometries may also, under suitable operative conditions, exhibit structural superlubricity. Concepts related to interface incommensurability and mechanical interlocking are still called into play when dealing with more complex mechanical contact configurations, as, for example, contacts between crystalline and amorphous surfaces^10,34^ and those realized during interwall dynamics in multiwalled nanotubes^42,72,73^, or in sliding and rolling of carbon nanotubes on substrates^74,75^, or even involving confined C_60_ molecules acting as nanoscale bearings^76^.

### Failure mechanisms and breakdown

**Fig. 4:**
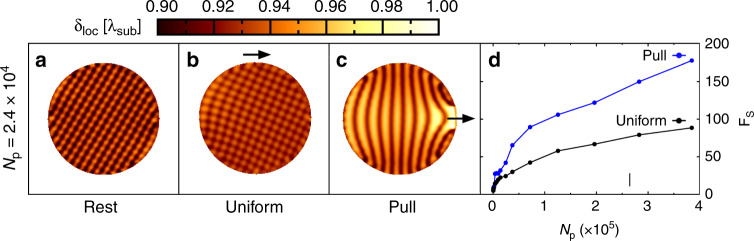
Strain distribution and static friction of a uniformly driven versus edge-driven 2D FK model. **a**–**c** Colored maps showing the local average distance δ_loc_ between nearest-neighbor particles in a 2D FK model of an incommensurate (overdense) circular island subjected to a periodic square-symmetry substrate potential^82^, 10.1103/PhysRevMaterials.2.046001. Expressed in units of the underlying periodicity, values of δ_loc_ = 1 (white color) indicate local commensuration to the substrate. The three panels compare the local strain distribution in the island at rest (**a**), and under the action of an external force, below the static friction threshold, applying a uniform driving (**b**) or pulling at the leading edge of the cluster (**c**). **d** Static friction force as a function of the contact area, i.e., the number of particles in the island, evaluated for uniform driving (black) and pulling at the leading edge. Due to the particle overdensity of the island with respect to the substrate minima arrangement, the edge pulling procedure favors the formation of locally strained commensurate regions, enhancing static friction.

Another intrinsic hindrance preventing immediate upscaling superlubricity comes from the mechanical contact elasticity at the slider/substrate interface. It has been theoretically anticipated that even for an incommensurate matching there exists a threshold size of contact, above which the elastic deformations may become sufficiently large to locally bring regions into a commensurate mating configuration (Fig. 4), leading thus to pinning and enhancement of friction^77–82^. This threshold depends on the ratio between the intra-surface and interfacial stiffness and on the imposed driving procedure. First, experimental verification of this prediction was reported recently for antimony particles sliding atop MoS_2_^83^. For 2D layered materials the effects of elasticity can become important already in microscale contacts. Undesirable elasticity effects can be diminished using multi-contact interfacial configurations, in which a macroscale junction is divided into a large collection of decoupled or weakly coupled, randomly oriented small-scale patches^84–86^. The elasticity effects can be also diminished by depositing the van der Waals material coatings on rigid substrates. This configuration prevents deformation of the contacting sliding layers therefore hampering the onset of local partially commensurate areas and reducing considerably frictional energy dissipation.

Under realistic conditions, where the contacts always have a finite size, the total friction is contributed not only by the surface atoms located within the contact area, but also by those at the contact edges^46^. Due to their low confinement, the edge bonds are more flexible than those located within the bulk of the contact area, and thus may undergo larger distortions during sliding^70,87^. Thus, the contact edges may act as pinning sites during sliding, enhancing frictional energy dissipation, and impeding superlubricity. Computational studies revealed that for incommensurate finite contacts, the edge atoms, more compliant to the action of the normal load, become easily pinned to the substrate^70,87,88^. In addition, under ambient conditions, exposure of edges to contaminations is inevitable, which also boosts frictional dissipation. Interestingly, these investigations demonstrated that at heterogeneous interfaces, e.g., between graphene and hexagonal boron nitride, the compliance and pinning of edge atoms under high normal loads is significantly reduced compared to the case of homogeneous graphitic contacts^70^.

Scaling up structural superlubricity toward the macroscopic world is further challenged by the often polycrystalline nature of layered materials at larger scales, where the contacting surfaces often exhibit a mosaic of randomly oriented grains that are separated by grain boundaries^89^. The grain boundaries typically induce large out-of-plane surface deformations that may degrade or even completely eliminate the superlubric behavior. Understanding the effects of edges and grain boundaries on the frictional dissipation, and the mechanical response in general, in layered material contacts is crucial for achieving large-scale superlubricity, but this information is still lacking, and it is hard to obtain from measurements with solid contacts and from simulations.

In addition to the discussed above intrinsic factors, which can suppress superlubricity, there are a number of external factors that can also hinder the superlubric regime. In this respect, it was found that a presence of contaminants, which are inevitable under ambient conditions, often leads to finite static friction and an increase of kinetic friction at the nominally incommensurate interface^34,90,91^. The contaminant-related friction may be diminished by surface heating, which leads to desorption of adsorbates^34,65,92,93^. Alternatively, in situ interface cleaning can be achieved by applying mechanical oscillations to the frictional contact, which can significantly reduce the concentration of contaminants and restore superlubric regimes^94,95^. Recent experimental and computational studies of structural superlubricity under ambient conditions suggested that contaminants are pushed out from the contact formed by mesoscopic gold islands and graphite, thus providing a robust ultralow friction state^36^. It is still not clear how general is the observed phenomenon, but the search for self-cleaning incommensurate contacts that repel contaminants may open up a possibility for realization of structural superlubricity under ambient conditions.

High normal loads applied to frictional contacts and high sliding velocities constitute other extrinsic factors limiting superlubricity. Normal loads and driving velocities explored in most friction measurements with nano- and microscale junctions of layered materials are significantly smaller than those usually applied in macroscopic tribological contacts. Obviously, increased friction and wear should be observed when the normal load and driving velocity exceed some threshold values that depend on the contact properties^96,97^. Therefore, it is important to determine the conditions under which the ultralow friction and wear are maintained for loads and velocities used in realistic applications.

If contact pressure may in general favor interface locking and friction, particularly acting on the compliant edge boundaries of a finite-size system, on the other hand, an increase in normal load can also suppress out-of-plane surface deformations, especially relevant for 2D layered materials, thereby inhibiting a dissipation channel and leading to a negative friction coefficient^98^. Most tribological systems show a logarithmic dependence of friction on the sliding velocity, which results from thermally activated crossing of sliding potential barriers^3^. Although such barriers are significantly suppressed in incommensurate contacts, the logarithmic velocity dependence of friction was also found in some superlubric systems^65,71^. This effect may originate from the contributions of edges, grain boundaries, and interfacial contaminants, however, more experimental and simulation studies are required to address this problem, which is important for both fundamental understanding of mechanism of superlubricity and for applications.

## Colloids and cold ions: limitless tribology emulators

### Precisely tunable experimental mesoscale systems

The ubiquity of friction and dissipative related processes, together with central correspondences regardless of the specific system and length scale, has recently stimulated novel experimental approaches and theoretical formulations capable of setting a common framework toward an understanding of these phenomena which is independent of the length scale and the specific choice of the involved materials. Such ongoing quest for the “holy grail” of a unifying approach to friction may undisputedly benefit from investigations at the small microscopic level, with sliding mechanical contacts now well-characterized and operating under well-designed experimental conditions.

In this scenario, the prediction of the system’s universal physical response is primarily based on interface geometrical features and relative interaction considerations, more than upon distinct system-dependent characteristics (such as the specific governing forces and their range). Accordingly, tribological experiments measuring how general parameters, e.g., substrate corrugation versus interlayer stiffness, lattice mismatch, shear velocities, and driving forces impact the frictional behavior, do become crucial. The most promising theoretical tool to understand system-independent general properties of friction are simplified ‘mechanical’ approaches^99,100^, such as the Prandtl–Tomlinson and the Frenkel–Kontorova type of models, relying on a firm fundamental background but whose direct experimental testing has been locked away for several decades.

**Fig. 5:**
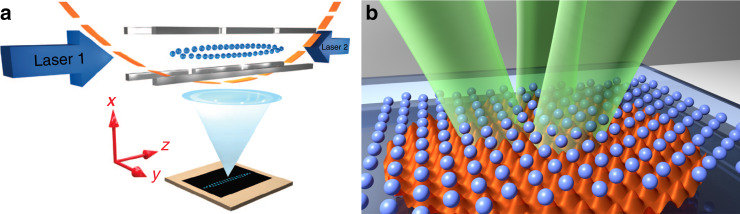
Experimental confirmation of the Aubry transition in atomic and colloidal systems. **a** Sketch of an experimental setup with two interacting chains of laser-cooled YB positive ions inside a linear trap which is created by four quadrupole electrodes. The two laser beams are used for the illumination of the ions and for performing vibrational spectroscopy. The ions are imaged onto an electron multiplying CCD camera^52^. **b** Illustration of an extended two-dimensional micron-sized colloidal monolayer consisting of several thousand particles which are interacting with an optical substrate potential created by three interfering laser beams. Variation of the corrugation amplitude of the substrate potential is achieved by changes in the laser intensity^53^. Both experiments confirm the existence of a transition from a superlubric to a pinned state in agreement with theoretical predictions.

In this view, out of serendipity, the field of microscopic tribology can now benefit from the experimental opportunities offered by subjecting nano/microparticles to artificial optical potentials, as shown in Fig. 5, or to topologically microstructured substrates, disclosing the possibility to directly visualize the detailed intimate mechanisms at play in sliding friction. Even though the role of inertia in atomic and colloidal systems is very different, this is irrelevant in context of structural superlubricity because a state of vanishing static friction only depends on the competition of length scales and energy frustrated configurations, but not on the way energy is dissipated at the interface. In addition to replicate many observations from atomic systems, the use of cold ions and colloids allows to tune several relevant physical parameters which are not easily accessible in hard matter systems^48,101^.

### System frictional response: unprecedented real-time insight into individual particle dynamics

While atomic force microscopy, surface-force apparatus, and quartz-crystal microbalance, workhorse techniques in experimental nanotribology, do measure the system rheological and dissipative response in terms of crucial, but averaged, physical quantities^102^, exploiting the versatility of these novel approaches has allowed to cast new light on elemental interface processes at the single-particle level with unprecedented real-time resolution, avoiding interpretative pitfalls arising from indirect or ex situ characterization of contact surfaces.

In order to resolve the single-particle dynamics of monolayers sliding across corrugated surfaces, it has been suggested to perform experiments with cold ions in electro-optical fields^103–105^. Such experiments would allow for the direct comparison with theory and numerical simulations of the Frenkel–Kontorova model which make detailed predictions regarding the motion on a single-particle level. Recently, experiments with laser-cooled ions have been accomplished where one-dimensional chains of ions were driven across periodically modulated potentials^51,52^, the latter provided, e.g., by spatially modulated electrical fields or another nearby chain of interacting ions. Apart from resolving the single-particle dynamics, this approach has another important advantage because it allows to vary the interaction between the sliding chain with the substrate in situ. This will be important, e.g., in the context of the Aubry transition as will be discussed further below.

Colloidal suspensions, i.e., micron-sized particles suspended in simple fluids, provide another approach to study nanotribological effects with high spatial and temporal resolution. Similar to atoms, they can form crystalline solids or glasses whose properties closely resemble those of their atomic counterparts^106^. Owing to the colloidal length scale and the presence of a viscous bath, however, their trajectories can be conveniently tracked in space and time with optical methods^107^. Compared to atoms and ions, interactions between colloids can be tailored, e.g., by functionalization of their surfaces with active end groups or by adding magnetic clusters during their fabrication process^108^. Therefore, colloidal pair interactions can vary between a short-ranged hard-sphere like behavior to more long-ranged forces as screened Coulomb or dipolar pair potentials. Similar to ions, colloids strongly couple to external fields offering the opportunity to taylor artificial substrates with a high degree of flexibility regarding the geometry, lattice spacing and importantly, also the strength of interaction^109–115^.

### From superlubricity to pinning: unraveling the nature of the Aubry transition in 1D and 2D systems

**Fig. 6:**
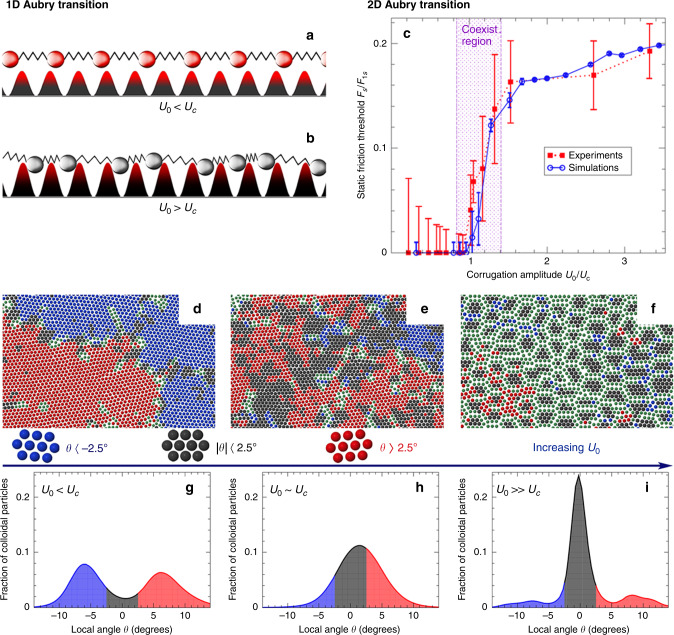
Aubry transition in 1D and 2D systems. **a**, **b** Schematic view of a 1D system of elastically interacting particles subjected to an incommensurate periodic substrate. **a** When the substrate corrugation amplitude U_0_ is below a critical value U_c_, particles are found at any displacement relative to substrate minima which results in a superlubric state. **b** When U_0_ > U_c_ particles become localized near the potential minima. This leads to a continuous Aubry transition from a superlubric to a pinned state. **c** Static friction force versus substrate corrugation obtained from experiments (solid symbols) and simulations (open symbols) of a 2D colloidal system; the shaded area shows the coexistence region, across which the Aubry transition takes place. Measured colloidal particle configurations for increasing substrate potential: **d** U_0_ < U_c_, **e** U_0_ ≈ U_c_, and **f** U_0_ > U_c_. The color code corresponds to the local misfit angle θ of the particles relative to the substrate. **g**–**i** Misfit angle-distribution for increasing substrate potential indicating the coexistence between a suberlubric and a pinned state^53^ in agreement with numerical simulations predicting a first-order Aubry transition in 2D^117,118^.

Mathematically proved roughly 40 years ago in a series of seminal papers addressing the fascinating complexity of spatially modulated structures, the Aubry transition (see, e.g., refs. ^24,25^ and references therein) stands nowadays at the core of the superlubricity phenomenon in frustrated incommensurate systems. In the framework of the infinite, 1D incommensurate FK model, where it was originally derived, the transition takes place at a critical value of the ratio between the substrate corrugation and the intra-chain stiffness (see Fig. 6a, b). Below the critical threshold, there exists a continuum of ground states for the chain (described by an analytical function) that can be reached through non-rigid displacements of its particles with no energy cost (goldstone mode); the sliding of the chain is initiated by even the smallest driving force and, accordingly, the static friction vanishes. Above the critical interaction ratio, on the contrary, the particles are trapped close to the minima of the substrate potential, and the two surfaces, locked together via local regions of common periodicity, do require a finite force to be mutually displaced. In the thermodynamic limit and at zero temperature, the Aubry transition of the 1D incommensurate chain-substrate interface exhibits a second-order character separating an unpinned superlubric regime from a pinned state. However, without the feasibility of freely tuning interaction parameters, as in the physics of layered materials, the nature of the Aubry transition cannot be clarified and bring to light how a change from a superlubric to a pinned state occurs.

Employing state-of-the-art experimental setups^51,52^, recently artificial frictional emulators consisting of finite 1D laser-cooled Coulomb ion crystals, have demonstrated Aubry-type signatures when set into motion across a periodic optical lattice under the action of an external electric field. By changing the structural mismatch between the ion chain and substrate, highly dissipative stick slip is tuned to a nearly frictionless dynamical state already at the level of just a few interacting particles^50^, revealing intriguing potential implications even into the quantum many-body regime^116^. Similarly, by effectively changing the mutual interaction strength within a setup of two deformable ion chains, the spatially resolved position of the trapped particles has revealed an Aubry structural phase transition between a free-sliding arrangement of the chain and a pinned fractal-like atomic configuration^52^.

While Aubry restricted his prediction to 1D contacts at zero temperature, recent experiments even demonstrate the validity of his ideas when considering extended 2D interfaces at room temperature (Fig. 6), which may be closer to realistic tribological conditions. Compared to the smoother nature of the original Aubry phenomenology in 1D, numerical simulations obtained for 2D contacts suggest a first-order transition from a pinned to a superlubric sliding state^117,118^. As a consequence, one expects a coexistence region between pinned and free-sliding patches which indeed has been recently demonstrated experimentally using colloidal monolayers on incommensurate surfaces^53^ (see panels (c–h) in Fig. 6). In addition, these experiments provide detailed insight into the microscopic origin of the Aubry transition, exhibiting for such compliant systems a strong structural feature correlated to the orientational misalignment, characterizing the majority of incommensurate interfaces^119^, of the adsorbed (colloidal) layer relative to the underneath (optical) substrate^120^.

In addition to colloidal monolayers, the Aubry transition may be also observed in other geometrically well-characterized soft-matter systems. However, it should be noted, that with increasing softness, additional effects such as lubrication may dominate their friction behavior^121^.

### Directional locking of particles on periodic surfaces

When single or weakly coupled (quasi)particles are driven across a substrate potential, they do not necessarily follow the direction of the applied force. Instead their direction of motion becomes strongly locked to the high symmetry axes of the substrate lattice. Evidence of such directional locking is found, e.g., in type II superconductors. When an external magnetic field penetrates the material a hexagonal lattice of localized flux bundles forms. In the presence of a lattice created by defects, the flux bundle motion shows a clear dependence on the direction of the driving force relative to the symmetry directions of the defect grid^122–124^. Similar observations have been obtained in colloidal systems forced over topographical or optical periodic landscapes. When an isolated colloidal particle is driven across a periodic surface with 4-fold rotational symmetry, its motion becomes strongly locked to the substrate which results in a devil’s staircase structure when plotting the direction of the particle motion vs. the angle of the external force^125,126^.

Directional locking can be understood by considering the corridors of lowest energy of a driven particle moving across the substrate potential^127,128^. Depending on the particle size and the lattice spacing, this results in corridors along the high-symmetry directions of the substrate and thus explains the above mentioned devil’s staircase hierarchy. Interestingly, the presence of thermal noise does not lead to the smoothing or even disappearance of the plateaus but to statistical jumps between different well-defined locking states^126,129^. In general, such locking-effects strongly depend on the interaction of the particles with the substrate. Accordingly, the phenomenon can be exploited for the sorting of colloids^130,131^, living cells^132^, and other macromolecules^132–134^.

Particle sorting by directional locking is not limited to spherical particles but has been also demonstrated in case of non-spherical objects. In particular for chiral particles, i.e., when the particle shape is not superimposable with its mirror image, efficient sorting mechanisms of both enantiomers (typically created in equal amounts during the synthesis) are important to separate, e.g., organic and inorganic chiral molecules whose pharmacological and toxicological effects may strongly depend on their chirality^135,136^. Numerical simulations demonstrate that when planar chiral particles are driven across a periodic substrate with square geometry, objects with opposite chirality move along orthogonal directions. In the presence of a periodic external driving force, even motion into exactly opposite directions can be achieved^137^.

Directional locking is not only affecting the direction of motion of single particles but can also affect the structure of a monolayer of interacting particles driven across a patterned surface. This has been demonstrated in case of electrostatically interacting colloidal particles driven across a quasiperiodic substrate potential with decagonal symmetry. Opposed to the situation where no external drive is applied and the particles follow the symmetry of the underlying substrate potential, numerical simulations and experiments demonstrate square, smectic, and also Archimedean-like states in the driven phases^112,138^.

**Fig. 7:**
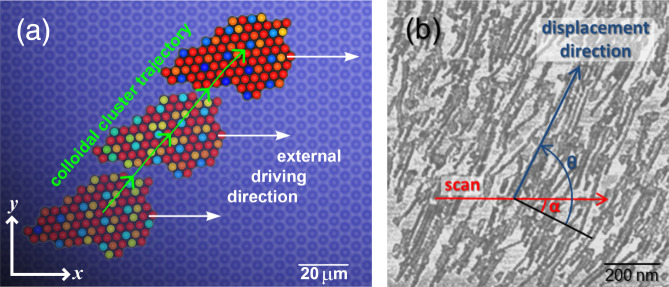
Directional and orientational locking of clusters driven across periodic surfaces. **a** Mesoscopic colloidal cluster whose direction of motion (green arrow) substantially deviates from that of the driving force (white arrow) due to the interaction with the underlying periodic substrate potential. In addition to the direction of motion the orientation of the cluster becomes locked during the sliding process. Particles are colored corresponding to their displacement relative to the nearest substrate potential well^142^. **b** AFM-manipulated crystalline gold nanoparticles on molybdenum disulfide also displaying a pronounced deviation between the direction of translation (blue arrow) versus external forcing (red arrow)^141^, 10.1103/PhysRevB.98.165417.

In addition to individual or weakly interacting particles^122,128,129,139^, directional locking is also observed in nanoscale manipulation and numerical simulations of Au-clusters on layered materials^140,141^ as well as in mesoscopic experiments with rigid clusters comprised of several tens of colloids^142,143^ (Fig. 7). Contrary to single particles, where the locking directions typically coincide with high-symmetry axes of the substrate, this is no longer true when the lattice spacing of the cluster is incommensurate with the substrate. Then, the center-of-mass motion becomes locked into directions entirely determined by the geometrical moiré superstructure formed by the cluster and substrate lattices. Because such moiré superstructures are not strictly periodic, this leads to competing locking directions depending on cluster size which strongly depend on the detailed shape of the surface corrugation profile. Apart from directional locking, the orientation of the driven clusters is also affected by the substrate. Because such phenomenology mainly depends on the lattice mismatch and cluster size, directional locking offers novel opportunities for the assembly of nano-objects in the presence of external driving forces.

## Conclusions

In this bird’s eye Perspective, we hope to have conveyed the flavor of an exciting research field, that of nano and microscopic friction and related out-of-equilibrium dissipative phenomena, which is alive and well.

With the interplay of experiments, theory and simulations, recent advances are currently pursued in a multifaceted approach, starting from the fundamental atomic-scale mechanisms at single-asperity level and then scaling up to interface phenomena in extended well-characterized nano and microscale contacts. Simultaneously, the novel exploitation of mesoscale tunable trapped cold ions and colloidal particles under shear is indeed going to shed light with unparalleled accuracy on several key tribological processes. Last but not least, noteworthy developments in synthesis and fabrication procedures allow now to extend the characterization of well-defined tribological interface, especially dealing with layered materials, up to the millimeter range and beyond, with direct possible implementation in the manufacturing of state-of-the-art mechanical devices. Unquestionably, a full understanding and control of frictional processes is now recognized to involve all relevant size and time scales.

The two lines of research outlined here, i.e., hard 2D layered materials and soft-matter systems, can indeed help to identify unexplored energy dissipation mechanisms and to provide comprehensive testing grounds for theoretical predictions with the overall aim to deepen our basic knowledge in this important discipline. The possibility to compare system- and scale-independent hard- and soft-matter interfaces brings about the unique chance to disentangle material-specific and geometrical aspects of the interfaces which allows a detailed view on friction-related phenomena and largely extends our basic knowledge in this field. Further studies in this direction can make a significant contribution to solving one of the most challenging problems in the field of tribology, i.e., bridging the scale gap between the microscopic and the macroscopic frictional mechanisms occurring at a sliding interface.

With their fundamental relevance toward practical engineering applications, the framework of 2D materials may strongly benefit from the synergic complementarity with the realm of soft tribology emulators, which offers the exceptional possibility to visually resolve the intimate nature of both structural features and dynamical mechanisms of sliding friction down to the individual particle level. Differently, the usual state-of-the-art tribological techniques provide experimental measurements typically in terms of crucial, yet averaged, frictional quantities, which make systematic inroads unfortunate when paired with the recognized difficulty of tuning the relevant physical parameters in condensed matter tribology.

Such “soft-hard” experimental synergy, together with modeling and numerical simulations, may thus contribute in drawing the roadmap to tackle the great scientific and practical challenge of addressing some aspects of the complex nature of frictional phenomena.

Setting the ground to robustly scale structural superlubricity up, transposing the desired peculiar nanoscale properties to larger tribological scales and recognizing the conditions of its persistence and the mechanisms of its breakdown, surely remains on the list of outstanding issues that have to be addressed thoroughly. With the aim of controlling frictional-induced energy dissipation in real-life sliding devices, inherent contact elasticity, grain boundaries, surface defects, and interface contamination are, and always will be, unavoidable key elements to consider when moving from the nanoworld up to the macroscopic domain. Besides, although some experiments have demonstrated wearless and superlubric motion for sliding distances of a few mm^71^, most microscopic measurements aiming at understanding basic tribological mechanisms are thus performed over short sliding distances. Wear is expected to be low but not completely eliminated even in the superlubric sliding regime. Thus, in order to address technological challenges, sustainability of superlubricity at long sliding distances, high normal loads, velocities, and temperatures must be experimentally studied.

Lively progress along these and newer lines is to be expected in the near future.
